# Prenatal exposure to environmental toxins and comprehensive dental findings in a population cohort of children

**DOI:** 10.1186/s12903-023-03786-2

**Published:** 2024-03-11

**Authors:** James R. Winkler, Barbara L. Dixon, Ishita Singh, Ray Soto, Yuqing Qiu, Yue Zhang, Christina A. Porucznik, Joseph B. Stanford

**Affiliations:** 1 School of Dentistry, University of Utah, Salt Lake City, UT USA; 2 Department of Pediatrics, University of Utah School of Medicine, Salt Lake City, UT USA; 3 Division of Public Health, Department of Family and Preventive Medicine, University of Utah School of Medicine, 375 Chipeta Way, Suite A, Salt Lake City, UT 84108 USA; 4 Division of Epidemiology, Department of Internal Medicine, University of Utah School of Medicine, Salt Lake City, UT USA

**Keywords:** Developmental defects in enamel, Molar incisor hypomineralization, Bisphenol-A, Phenols, Fluoride, Children

## Abstract

Environmental toxins are known to have many impacts on growth and development in humans, starting in utero. Alterations in amelogenesis, caused by chemical and physical trauma that occur during the antenatal, perinatal and postnatal time periods, may result in developmental defects in deciduous and permanent tooth enamel, as demonstrated in animal studies. These defects can be clinically visible and result in a variety of morphological and functional problems in the dentition. Since enamel does not remodel after formation, it may serve as a permanent record of insults during organ development.

Our primary purpose was to investigate any possible relationship between intrauterine exposure to endocrine disrupting chemicals (phenols and phthalates) and developmental defects in enamel in children, while also accounting for fluoride exposure. Our secondary purpose was to report descriptively on findings from comprehensive dental examinations performed on 356 children that were drawn from the general paediatric population. A cohort of children from the Utah Children’s Project (N = 356) that had full medical exams, comprehensive medical and family histories and available biospecimens were given extraoral and intraoral examinations. They also completed an oral health questionnaire. Standardized intraoral photographs were taken of the teeth and viewed by standardised examiners and the dental observations were recorded for a full inventory of findings, including: tooth morphology, caries, restorations, colorations, attrition, erosion, fractures and hypomineralization. Perinatal maternal urine samples were assessed for the concentration of fluoride, phenols and phthalates, including bisphenol A (BPA).

Pairwise statistical analyses were done to correlate the dental findings with one another and with the presence of environment chemicals found in the urine samples. Hypomineralization was the most common finding (96% of children; 37% of deciduous teeth, 42% of permanent teeth), consistent with molar incisor hypomineralization (MIH) described in other human populations. No consistent correlations were seen between dental findings and the presence of phenols and phthalates in prenatal urine, but the number of samples available for the assessment was limited (n = 35).

In conclusion, we found a high proportion of dental hypomineralization in a population based paediatric cohort, but did not find an association with prenatal exposure to phenols and phthalates.

**Supplementary Information:**

The online version contains supplementary material available at 10.1186/s12903-023-03786-2.

## Introduction

A wide range of environmental exposures that occur around the time of human conception and during sensitive developmental windows in pregnancy, infancy and early childhood can have lasting effects on the health and well-being of children. [1] In 2016, the National Institutes of Health (NIH) launched an initiative called *Environmental Influences on Child Health Outcomes* (ECHO). This consortium uses existing paediatric cohorts and supports multiple, synergistic, longitudinal cohort studies and clinical trials performed on mothers and children nationwide. [2]

As part of the ECHO consortium, the University of Utah contributes a paediatric cohort known as the Utah Children’s Project (UCP). [3] This group is comprised of over 500 proband children and 400 siblings derived from over 500 families, who were previously enrolled in several preconception or prenatal cohort studies (described further below), so that data were available for the proband children from the perinatal and/or preconception time periods. The UCP has performed full phenotyping examinations on children and parents, completed comprehensive medical and family histories, collected biospecimens and assessed a variety of early environmental exposures. In a subset of UCP children, maternal periconceptional and prenatal urine samples are available.

Addressing the health of children comprehensively necessitates inclusion of their dental health. There is growing interest in the correlation of environmental exposures and dental health indicators. [4, 5] Some alterations in enamel development change the normal translucence of teeth. [6] A major group of alterations is categorized as Developmental Defects of Enamel (DDE). [7] These changes are seen clinically as hypoplasia, hypomineralization, demarcated opacities, diffuse opacities and mottling, or some combination thereof. [8] Animal models have shown a strong positive correlation between environmental toxins and Development Defects in Enamel (DDE), such as Molar-Incisor hypomineralization (MIH) and endocrine disruptors, such as Bisphenol A (BPA). [9]

The primary purpose of this investigation was to investigate any possible relationship between intrauterine exposure to endocrine disrupting chemicals (phenols and phthalates) and developmental defects in enamel (DDE) in children, while also accounting for any possible role of fluoride exposure. We hypothesized that prenatal exposure to BPA or other phenols or phthalates may correlate to DDE in humans. [9, 10] The secondary purpose of this investigation was to report descriptively on findings from comprehensive dental examinations performed on 356 children that were drawn from the general paediatric population in Utah (i.e., UCP participants).

## Methods

### Utah children’s project dental cohort

A subset of children from the UCP were identified based on their prior participation in the Utah sites of the National Children’s Study within Salt Lake and Cache Counties, a population-based cohort of children born between 2009 and 2012, or from the Home Observation of Periconception Exposures (HOPE) study, which recruited volunteers from the greater Salt Lake City metropolitan area born between 2011 and 2015, and one of their siblings. [11, 12] A dedicated follow-up visit for dental evaluation was scheduled for children from these cohorts who were age 4 or more at the time of the scheduled visit. Visits were conducted between December 2016 and February 2020, creating a dental cohort of patients. The dental cohort consisted of 356 children (210 probands and 146 siblings) born from 216 mothers. After informed consent was obtained, an oral health questionnaire was administered documenting the history and status of the child’s dentition, fluoride supplements and applications, dental procedures performed, the frequency and nature of visits to a dentist and antibiotic usage by the mother during and following pregnancy for each participant in the dental cohort.

The data obtained were added to each subject’s study information that included geographic and demographic data, such as age, gender, race, ethnicity and a comprehensive personal and family medical histories. We used the STROBE checklist, version 4, in preparing this report.

### Clinical examination

Every child received an extra-oral and intra-oral examination of the hard and soft tissues by an oral health care provider that included palpation of soft tissues and visual evaluation of hard and soft tissues. Examination procedures were standardized by training and observation by senior dental clinician researchers (JRW and RND). Digital photographs were taken using a Cannon EOS Rebel T6 camera, Cannon EF 100 mm f/2 USM medium telephoto lens with ring flash. Direct or mirror view photographs were taken of the mandible and maxilla including occlusal, buccal, labial and palatal surfaces. The clinical observations and digital photographs were then added to the patient’s electronic study record.

### Dental finding analyses

To allow for standardization and in-depth analyses, the masked digital photographs were visualized on high-definition computer screens in a darkened room and evaluated independently by three unbiased and calibrated examiners (JRW, IS, BLD). [13] The number of clinically visible (fully or partially erupted) deciduous and permanent teeth were enumerated.

The dental findings for each tooth were recorded: visible restorations (amalgams, tooth coloured composites), sealants/preventive resin restorations, tooth coloured crowns and stainless steel crowns, hypomineralization, caries, occlusal/cuspal dysmorphologies, enamel spurs, discolorations, attrition/wear, erosion and fractures. The observations were recorded using a Microsoft Excel (2020) spreadsheet for all teeth and surfaces including the anterior incisal or posterior occlusal, cusp tips (buccal and lingual) as well as the buccal and lingual surfaces. The location of where on the tooth the finding was observed was also noted (incisal/occlusal 1/3, middle 1/3 and cervical 1/3). For the analyses in this paper, the dental conditions observed were grouped into the following categories: pit and fissure caries, smooth surface caries, discoloration, general wear, restorations, sealants, dysmorphogenesis and hypomineralization.

### Urinalyses for fluorides, phenols and phthalates

For a subset of dental cohort participants (N = 25), periconceptional maternal urine samples were previously self-collected by the proband’s mother at home and frozen in liquid nitrogen (LN2) for future analyses. Samples of this urine were sent to NMS Labs, Horsham, PA for fluoride analysis by ION Specific Electrode analysis (reporting limit: 0.20 mg/L).

Another subset of urine samples (some overlapping with the samples above) from 35 proband mothers were collected during the preconception or prenatal period and frozen (-80 C freezer). Aliquots were sent to the CHEARS Wadsworth laboratory and analysed for a panel of phenol and phthalate metabolites, including: Bisphenol A (BPA), Bisphenol S (BPS), Bisphenol F (BPF), Benzophenone-3(BP-3), Benzophenone-1 (BP-1), 2,4-Dichlorophenol(2,4-DCP), 2,5-Dichlorophenol (2,5-DCP), 2,4,6-Trichlorophenol 2,4,6-DCP, Trichlorosilane (TCS), Triclocarbin (TCC), 5-ethyl-2-methylpyridine (MeP), ethylparaben (EtP), propyl paraben (PrP), iso-butyl paraben (BuP) and benzyl paraben (BzP). [14]

### Statistical analyses

The spreadsheet used for data collection was converted into a SAS data set. Data management and analyses were performed using both SAS 9.4 and R version 3.6.3 (2020-02-29). We first used descriptive statistics to summarize the distributions of demographic characteristics (i.e. age, gender, race/ethnicity) and a variety of dental conditions by participant child. We also summarized the distributions of dental findings by the type of tooth (i.e. deciduous vs. permanent). The percentage of hypomineralization was summarized among molar tips and incisor tips, respectively, by the type of tooth, and also across all teeth and by the type of tooth were calculated. From these data, we calculated the Molar Incisor Hypomineralization Index (MIH), as done in prior studies. [13, 15] We calculated the Spearman correlation coefficients between the number of teeth with MIH and the prevalence of various dental health conditions. We generated a heat map to summarize the pair-wise Pearson correlation coefficients between different phenols and phthalates in the maternal urine samples. We generated graphs and calculated correlation coefficients for the proportion of teeth with lesions or hypomineralization with levels of environmental phenols (including BPA) and phthalates exposures measured in the periconceptional maternal urine samples.

## Results

### Demographic data

Demographic data were available for 355 of the 356 probands and siblings. The gender distribution was 47% female and 53% male. The age range was from 3 to 19 years of age (Mean 7.3; Median 7) at the time of examination. The population self-identified themselves as 89% white of non-Hispanic or Latinx origin, 6% Hispanic or Latinx, with the remainder not responding or unknown.

### Dental conditions in the participants

At the participant level (N = 356), dental conditions for all teeth present and visible were calculated (Table 1*)*. Over 95% of the subjects had one or more visible lesions. Hypomineralization was the most frequently seen dental condition (95.8%). The next most common conditions were occlusal, cusp and incisor wear (87.9%), pit and fissure caries (42.7%), restorations (41.6%), sealants (32.6%) and smooth surface caries (9.0%). Dysmorphogenesis, as assessed by a gnarled occlusal morphology or smooth surface defects (bumps), was the least common dental condition observed (3.9%).

**Table 1:** Dental findings by participant child (n = 356) Dental Finding N (%)

Pit and Fissure Caries	152 (42.7)
Smooth Surface Caries	32 (9.0)
Discoloration	59 (16.6)
General Wear	313 (87.9)
Restorations	148 (41.6)
Sealants	116 (32.6)
Dysmorphogenesis	14 (3.9)
Hypomineralization	341 (95.8)

### Tooth level dental conditions

Individual tooth analyses were done to determine whether the conditions occurred in the deciduous or permanent teeth (Table 2). Hypomineralization was commonly seen in the permanent dentition (41.7%) and seen slightly less frequently in the deciduous dentition (36.5%). Tooth level analyses revealed wear predominantly in the deciduous teeth (44.8%) and, to a much lesser extent, in the permanent teeth (2.6%). All forms of caries recorded were more frequently seen in the deciduous teeth (6.5%) compared to permanent teeth (1.4%). Dysmorphogenesis and discoloration were uncommon in the permanent and deciduous teeth.

**Table 2:** Dental findings by tooth

	Deciduous N (%)	Permanent N (%)	Total N (%)
Pit and Fissure Caries	302 (5.4%)	28 (1.3%)	330 (4.3%)
Smooth Surface Caries	61 (1.1%)	2 (0.1%)	63 (0.8%)
Discoloration	135 (2.4%)	26 (1.2%)	161 (2.1%)
General Wear	2501 (44.8%)	55 (2.6%)	2556 (33.2%)
Restorations	468 (8.4%)	44 (2.1%)	512 (6.7%)
Sealants	44 (0.8%)	217 (10.3%)	261 (3.4%)
Dysmorphogenesis	16 (0.3%)	9 (0.4%)	25 (0.3%)
Hypomineralization	2049 (36.5)	879 (41.7%)	2919 (38.0%)

## Prevalence of hypomineralization on molar cusps and incisor tips and MIH index

The percentages of hypomineralization seen on the permanent and deciduous molar cusp tips and incisal edges are shown in Table 3. Similar percentages of hypomineralization were present on permanent and deciduous molar tips (61.9% and 60.4% respectively) with a combined percentage of 61.6%. However, in permanent incisor tips, hypomineralization (28.5%) was seen more than twice as frequently than on deciduous incisor tips (11.8%), resulting in a combined percentage of 18.4%.

The percentages of molar cusp tips and incisor tips for permanent and molar teeth using the twelve MIH index teeth (the permanent first molars and the permanent incisors) were calculated (Table 3), resulting in a MIH Index of 41.3%. [16]

**Table 3:** Prevalence of hypomineralization on molar cusps and incisor tips and molar incisor hypomineralization (MIH) index

**Molar tips**		
Deciduous N = 2639	Permanent N = 717	Total N = 3356
1634 (61.9%)	433 (60.4%)	2067 (61.6%)
**Incisor tips**		
Deciduous N = 1649	Permanent N = 1072	Total N = 2721
194 (11.8%)	306 (28.5%)	500 (18.4%)
**MIH index**		
Deciduous N = 4288	Permanent N = 1789	Total N = 6077
1828 (42.6%)	739 (41.3%)	2567 (42.2%)

### Correlation between MIH and clinical findings

**Fig. 1:**
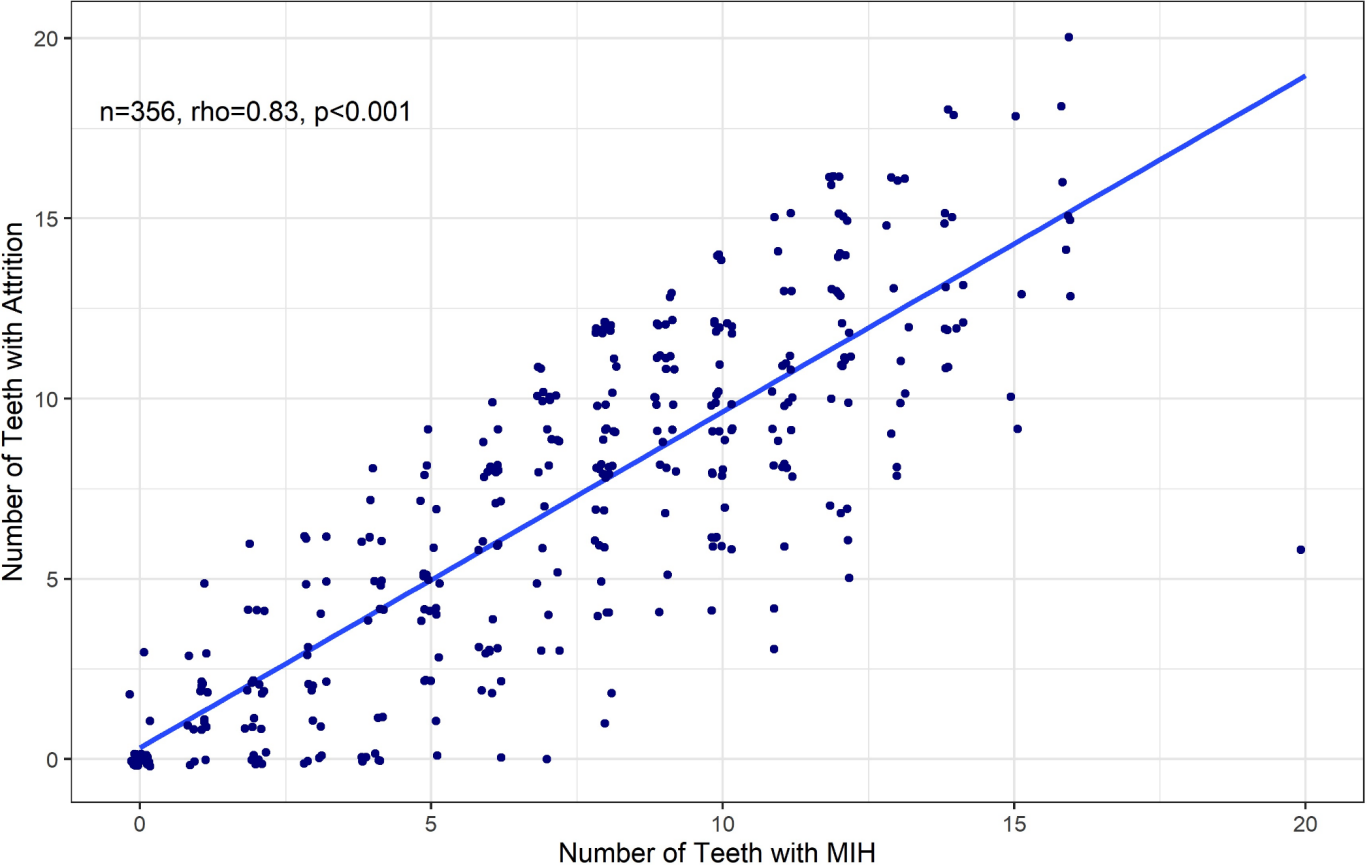
Correlation Between Wear and Teeth with MIH, Number of Affected Teeth per Participant. Each participant has one point on the figure, which correlates number of teeth with MIH with the number of teeth demonstrating attrition

Weak to no correlations were seen for pit and fissure caries, smooth surface caries, discoloration and dysmorphogenesis, in relation to MIH. Slightly positive correlations were seen for restorations (rho = 0.39, p < 0.001) and sealants (rho 0.26,p < 0.001) (Data not shown). However, as seen in Fig. 1, molar cusp wear and incisal wear (attrition) showed a strong correlation to the presence of MIH (rho = 0.83, p = 0.001).

### Maternal urine analyses for fluoride, phenols and phthalates

**Fig. 2:**
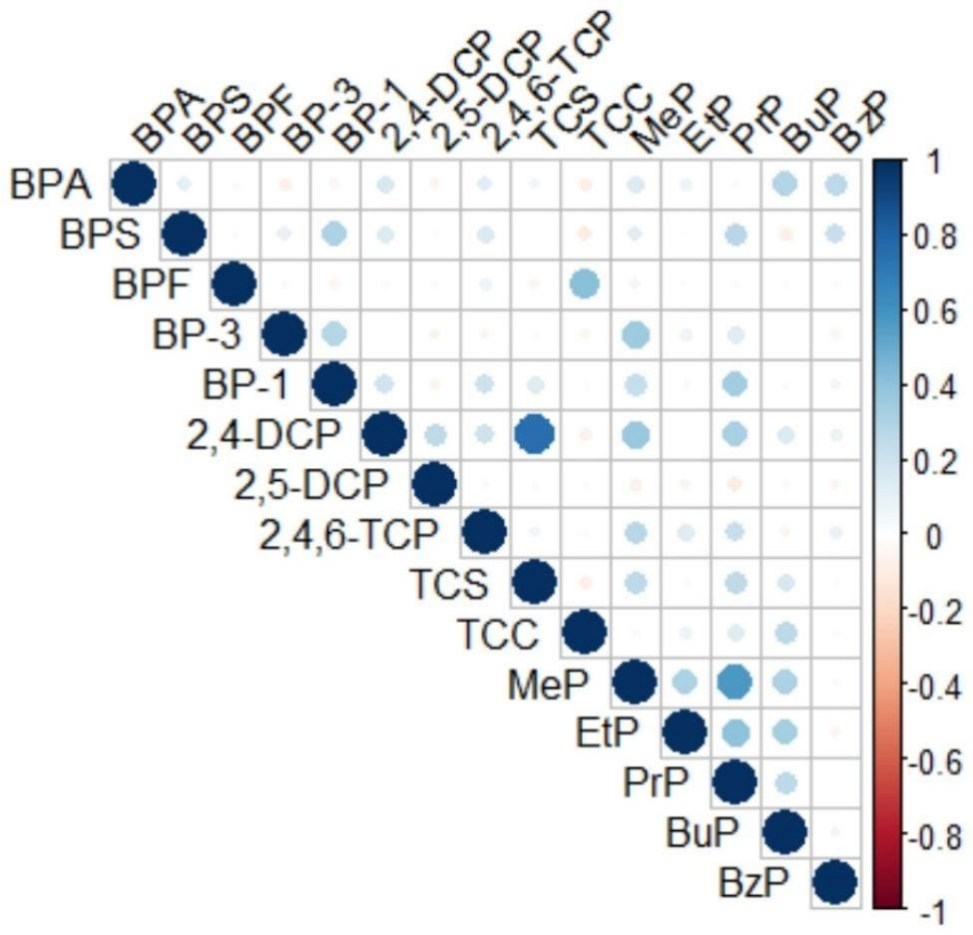
Pairwise correlation matrix of phenols and phthalates within periconceptional or prenatal urine samples (n = 35)

The 25 urine samples analysed for fluoride showed a concentration with an average of 0.73 mg/L (SD +/- 0.27, Median 0.72). A correlation matrix for the 35 samples analysed for phenols and phthalates showed that 2,4-DCP and TCS were the only chemicals that were significantly colinear in their urine concentrations (Fig. 2).

#### Correlation of chemicals in maternal urine and child dental findings

Weak positive correlations were found between increasing concentration of BPA, BP-1, EtP, and PrP and increased number or proportion of teeth with hypomineralization, while the 11 other chemicals had a weak positive association or no association. None of the associations were statistically significant. On the other hand, there was a statistically significant positive correlation between the maternal urine concentration and the number or proportion of teeth with any type of lesion for BPS and BuP; and a statistically significant inverse correlation for BPA. (*Appendix Figures*)

## Discussion

While hereditary DDE are relatively rare, there are many possible environmental causes of DDE. These may include trauma, systemic diseases, nutrition, fluoride, antibiotics, toxins and transient endocrine disrupting chemicals, such as phthalates and phenols. [17–25] Such insults impact the quality and/or quantity of enamel and dentine and can range in severity, depending on the extent and the duration of when the insult occurs to the enamel organ. [8, 26] Acquired DDE have been reported in 10–40% of primary teeth and in 9–63% of permanent teeth in healthy children in advanced countries. [27] While DDE are commonly encountered in practice, they are frequently misdiagnosed or overlooked. [28, 29] Clinically, DDE create retentive and irregular sites for cariogenic plaque to accumulate; make the enamel less resistant to bacterial and ingested acids; and cause the enamel to be thinner weaker and more susceptible to wear and erosion. [30, 31]

In our dental cohort, we found that a large percentage of teeth had a distinct pattern of hypomineralization, including the molar cusp tips and the incisal 1/3 of the permanent incisors. This finding is consistent with a diagnosis of molar-incisor hypomineralization. [16] MIH was initially described as a non-fluoride DDE showing this distinctive pattern for molars and incisors. [32, 33] However, it is now known that all permanent and deciduous teeth may be affected as well, similar to what we observed in our dental cohort. [13, 34] The aetiology of MIH appears to be multifactorial and may be due to systemic factors, such as acute or chronic illnesses or exposures to environmental pollutants during the last gestational trimester and first three years of life. [35, 36] With our dental cohort, we are not able to identify the root aetiology of the MIH, although we investigated several possibilities.

The urine fluoride levels we analysed in prenatal urine were within the normal range. Although number of prenatal and periconception urine samples we analysed was relatively small, we believe there is no reason to expect the fluoride findings would differ with a greater sample size. The geographic analyses of the fluoride in the water supplies for this dental cohort (data not shown) are optimal to sub-optimal and would not contribute to the extensive hypomineralization seen. The medical and dental histories revealed that mothers of the participants did not use prenatal fluoride drops. We are confident that our findings are not due to overexposure to fluoride. We also did not find a history of antibiotic usage or other drug usage in pregnancy to account for our findings.

BPA, a known endocrine disruptor, is found in a wide range of products and is widely distributed in the environment, and has been shown to have many adverse effects on humans. [37, 38] BPA has been implicated as a causative agent of MIH-like lesions in rats. [9] Chronic exposure to BPA can also exacerbate dental fluorosis in growing rats. [39] However, when BPA is administered to rats with a combination of other endocrine disruptors, the effect on enamel is less severe than when administered alone. ^[25]^ In our analyses, there was a weak correlation between BPA and MIH, but it was not statistically significant. Additionally, we did not find consistent relationships between the additional phenols and phthalates measured and any of the dental findings in our dental cohort. Our findings were limited by the small number of urine samples we had available for analyses. It’s important also to note that a single urine sample does not always correctly classify ongoing exposure for chemicals with a short half-life. [40] Blood and urine analyses of maternally-derived specimens during early to late stages of pregnancy provide only a cross-sectional view of foetal exposures to toxins and chemicals. [5, 41]

The percentage of MIH we found in our dental cohort was high compared to other studies, which have reported prevalence from 2.4 to 40.2%. [42] This variability is likely due to the lack of standardized tools to diagnose MIH, which has most likely led to underestimation. Data collection instruments have been developed to help facilitate standardized data collection in epidemiological studies on MIH. [43] The MIH/HSPM index developed by includes hypomineralized second primary molars (HSPM) as index teeth, since they form at a similar time as the first permanent molars. [44]

The strong relationship between the number of teeth showing signs of wear and the teeth with hypomineralization is consistent with the pathophysiology and prior research findings. Alterations in MIH enamel make it more susceptible to wear and erosion, caries and restorations. MIH in humans can have long-term clinical implications for short and long-term management of the dentition. These implications include an increased risk of caries, and dental hypersensitivity, which is sometimes associated with dental anxiety, and can also contribute to poor oral hygiene. [15] Early diagnosis of MIH may facilitate prevention and treatment strategies for these conditions.

Our hypothesis that environmental toxins, such as BPA, may be responsible for the MIH seen in this cohort, was not confirmed by our findings, but the available number of prenatal urine samples was limited. Further research to identify environmental insults that may be leading to MIH is warranted in our population and other populations around the world.

At this time, it is not clear why this high level of MIH was seen in our dental cohort. Further studies are required to understand the high MIH seen in the participants in this group of Utah children and to investigate the role environmental toxins may play in the growth and development of children around the world. On the public health level, our findings reinforce the need for comprehensive and timely access to appropriate dental care in all paediatric populations.

## Conclusions

In this Utah paediatric dental cohort, most patients showed one or more visible enamel defects in their dentition. Hypomineralization was the most common finding affecting a significant number of deciduous and permanent teeth and was associated with dental attrition. Our sample size was limited to assess any relationship between exposures assessed in maternal urine and the child dental findings. Future studies are required to determine whether phenols and phthalates and/or other environmental factors are responsible for or contribute to the DDE findings in these and other children.

### Electronic supplementary material

Below is the link to the electronic supplementary material.

**Supplementary Material 1: Supplemental Figure 1.** Correlation between bisphenol A (BPA) in maternal urine, and numbers of teeth with dental lesions, numbers of teeth with hypomineralization, percentage of teeth with hypomineralization, and percentage of teeth with lesions. Each dot represents one child/mother dyad

## Data Availability

Data from this study is obtainable from the Drs. Stanford and Porucznik upon request and with appropriate IRB review.
