# Supplementary material for: Prenatal exposure to environmental toxins and comprehensive dental findings in a population cohort of children
Source: BMC Oral Health. 2024 Mar 11;24:326. doi: 10.1186/s12903-023-03786-2 (PMC10929125; doi:10.1186/s12903-023-03786-2)
Supplement: Supplementary file 1 — Supplementary Material 1: Supplemental Figure 1. Correlation between bisphenol A (BPA) in maternal urine, and numbers of teeth with dental lesions, numbers of teeth with hypomineralization, percentage of teeth with hypomineralization, and percentage of teeth with lesions. Each dot represents one child/mother dyad [file 12903_2023_3786_MOESM1_ESM.docx]

**Supplemental Figure 1.**


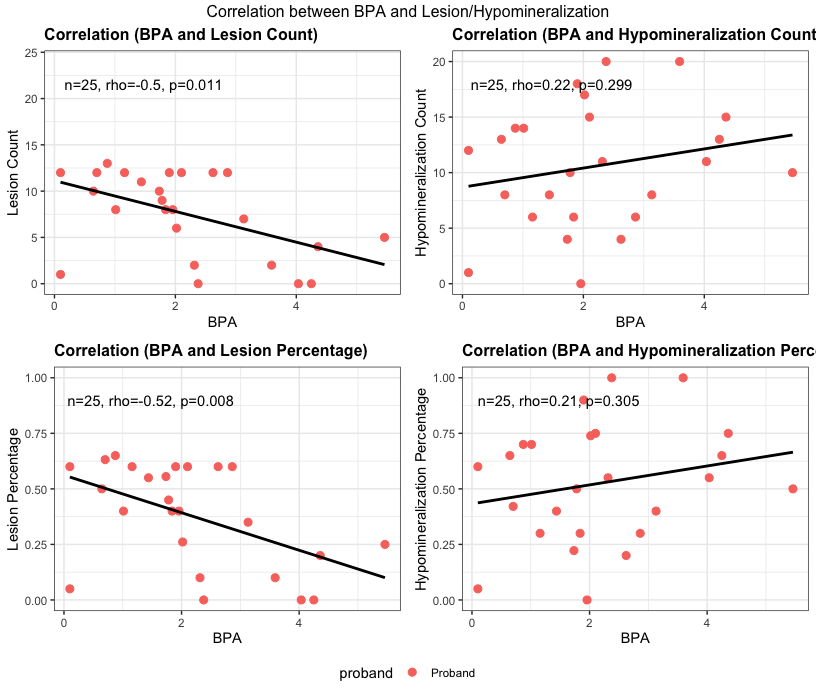


**Supplemental Figure 1. Correlation between bisphenol A (BPA) in maternal urine, and numbers of teeth with dental lesions, numbers of teeth with hypomineralization, percentage of teeth with hypomineralization, and percentage of teeth with lesions. Each dot represents one child/mother dyad.**

**Supplemental Figure 2.**


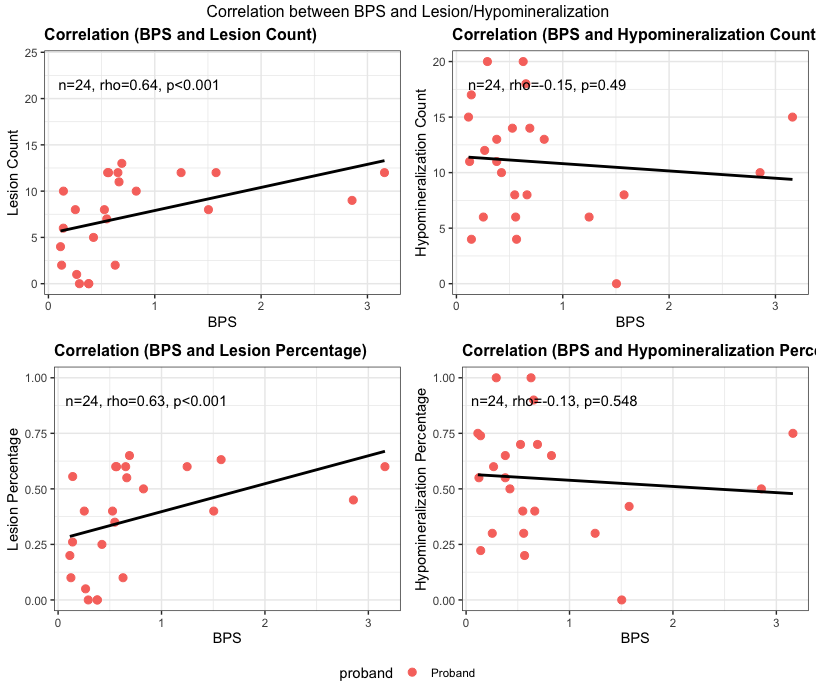


**Supplemental Figure 2. Correlation between bisphenol S (BPS) in maternal urine, and numbers of teeth with dental lesions, numbers of teeth with hypomineralization, percentage of teeth with hypomineralization, and percentage of teeth with lesions. Each dot represents one child/mother dyad.**

**Supplemental Figure 3.**


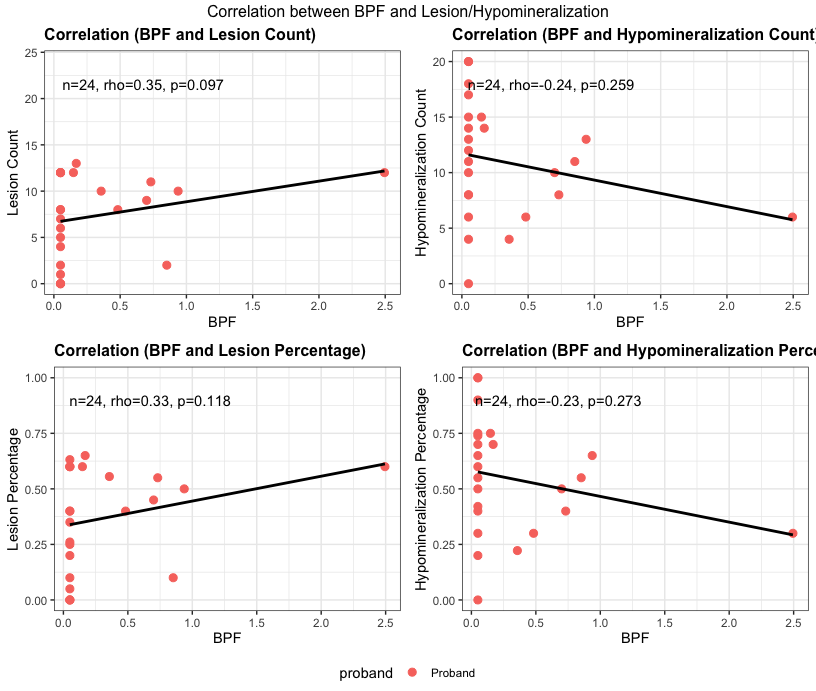


**Supplemental Figure 3. Correlation between bisphenol F (BPF) in maternal urine, and numbers of teeth with dental lesions, numbers of teeth with hypomineralization, percentage of teeth with hypomineralization, and percentage of teeth with lesions. Each dot represents one child/mother dyad.**

**Supplemental Figure 4.**


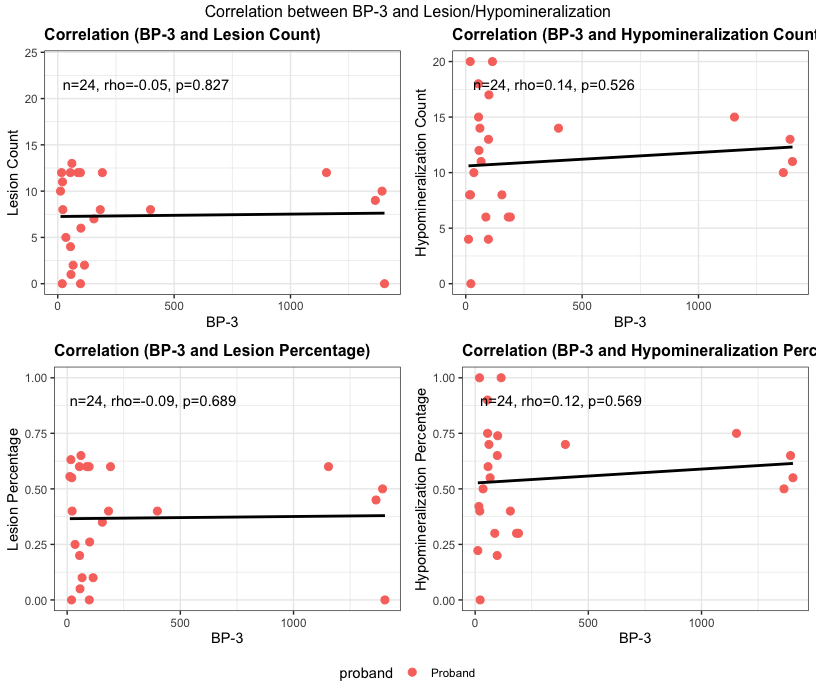


**Supplemental Figure 4. Correlation between benzophenone 3 (BP-3) in maternal urine, and numbers of teeth with dental lesions, numbers of teeth with hypomineralization, percentage of teeth with hypomineralization, and percentage of teeth with lesions. Each dot represents one child/mother dyad.**

**Supplemental Figure 5.**


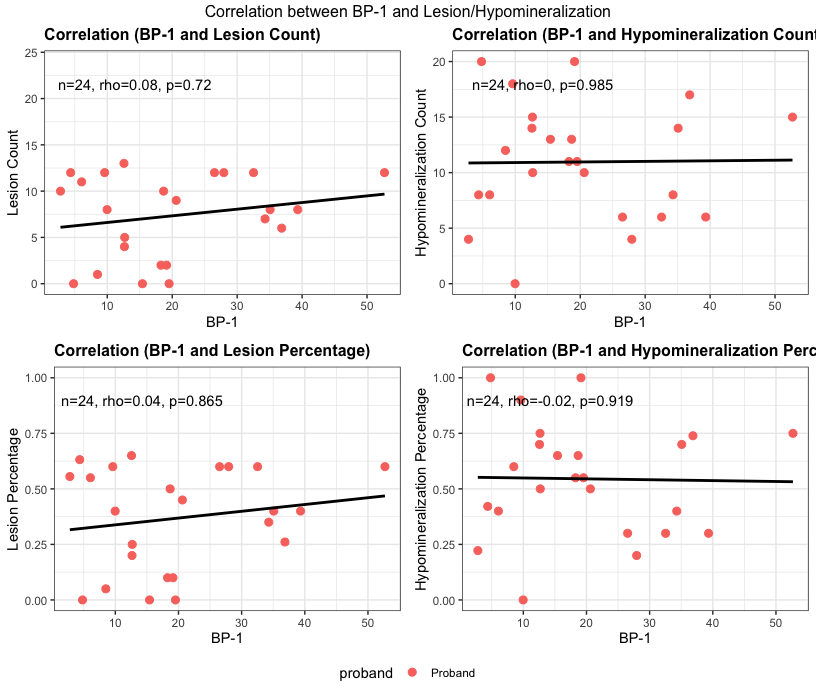


**Supplemental Figure 5. Correlation between 2,4-dihydroxybenzophenone (BP-1) in maternal urine, and numbers of teeth with dental lesions, numbers of teeth with hypomineralization, percentage of teeth with hypomineralization, and percentage of teeth with lesions. Each dot represents one child/mother dyad.**

**Supplemental Figure 6.**


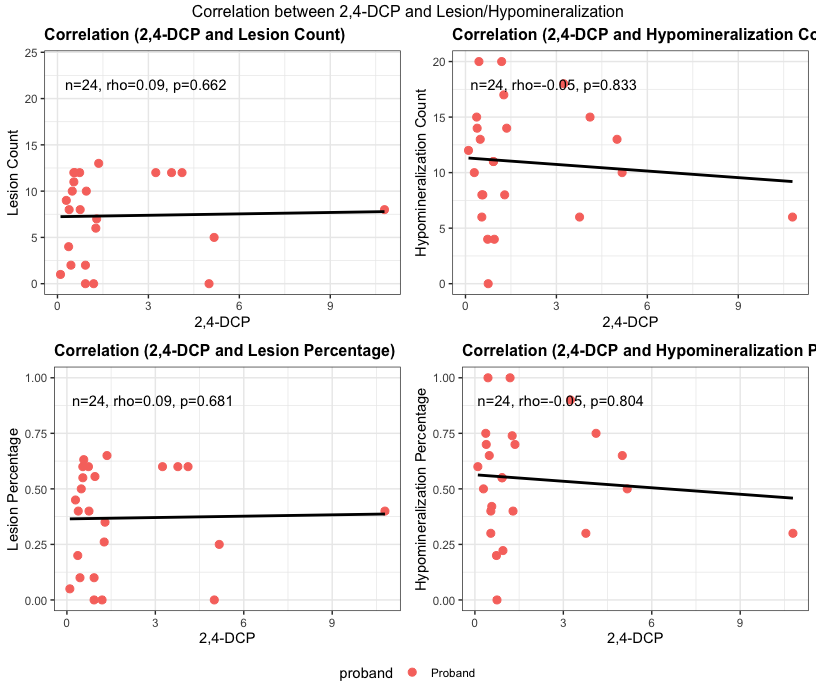


**Supplemental Figure 6. Correlation between 2,4-dicholorphenol (2,4-DCP) in maternal urine, and numbers of teeth with dental lesions, numbers of teeth with hypomineralization, percentage of teeth with hypomineralization, and percentage of teeth with lesions. Each dot represents one child/mother dyad.**

**Supplemental Figure 7.**


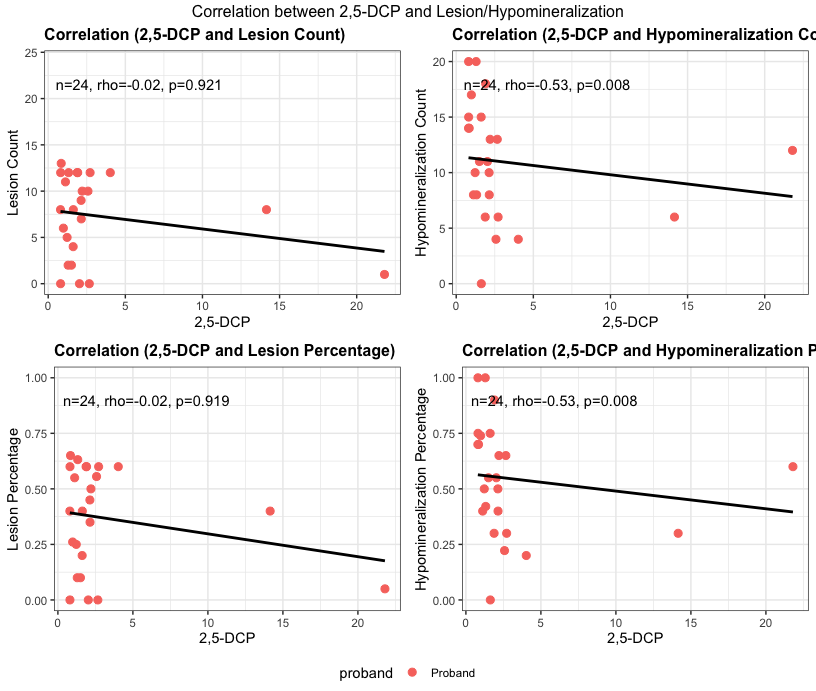


**Supplemental Figure 7. Correlation between 2,5-dicholorphenol (2,5-DCP) in maternal urine, and numbers of teeth with dental lesions, numbers of teeth with hypomineralization, percentage of teeth with hypomineralization, and percentage of teeth with lesions. Each dot represents one child/mother dyad.**

**Supplemental Figure 8.**


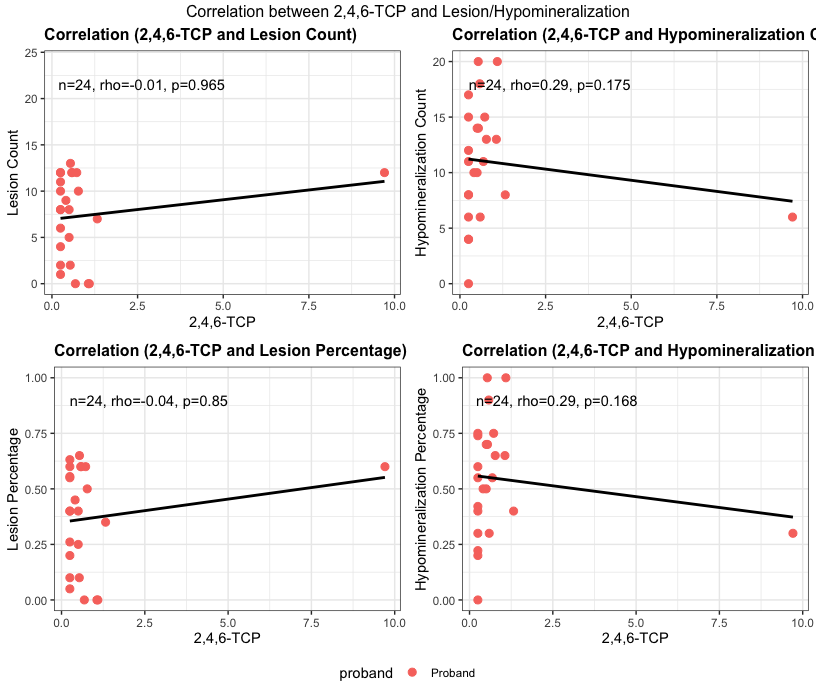


**Supplemental Figure 8. Correlation between 2,4,6-tricholorphenol (2,4,6-TCP) in maternal urine, and numbers of teeth with dental lesions, numbers of teeth with hypomineralization, percentage of teeth with hypomineralization, and percentage of teeth with lesions. Each dot represents one child/mother dyad.**

**Supplemental Figure 9.**


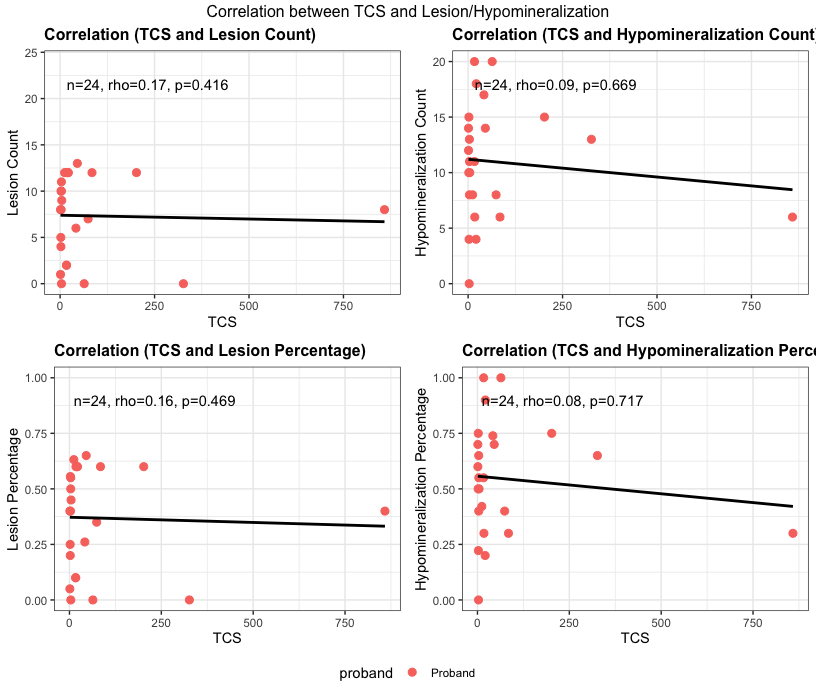


**Supplemental Figure 9. Correlation between triclosan (TCS) in maternal urine, and numbers of teeth with dental lesions, numbers of teeth with hypomineralization, percentage of teeth with hypomineralization, and percentage of teeth with lesions. Each dot represents one child/mother dyad.**

**Supplemental Figure 10.**


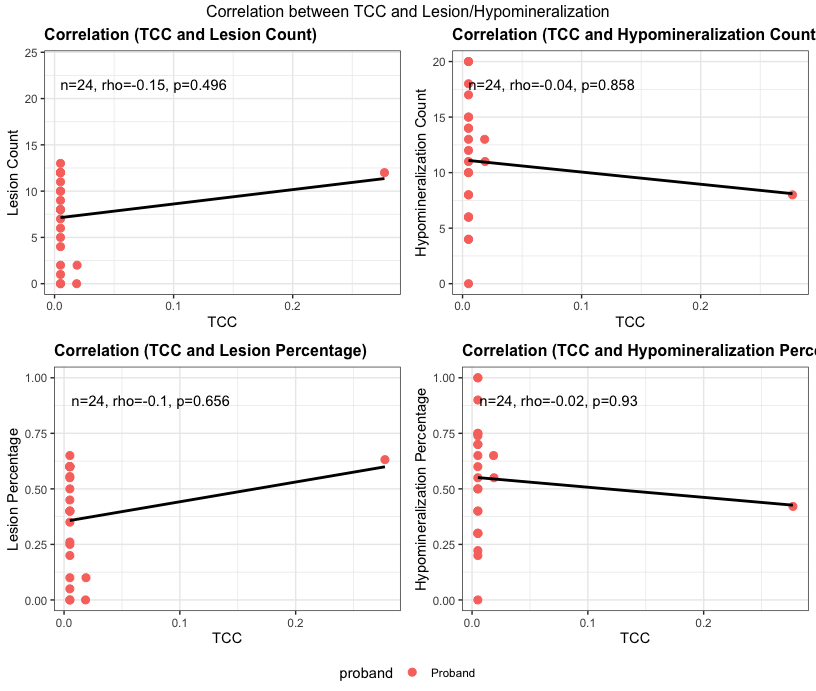


**Supplemental Figure 10. Correlation between triclocarban (TCC) in maternal urine, and numbers of teeth with dental lesions, numbers of teeth with hypomineralization, percentage of teeth with hypomineralization, and percentage of teeth with lesions. Each dot represents one child/mother dyad.**

**Supplemental Figure 11.**


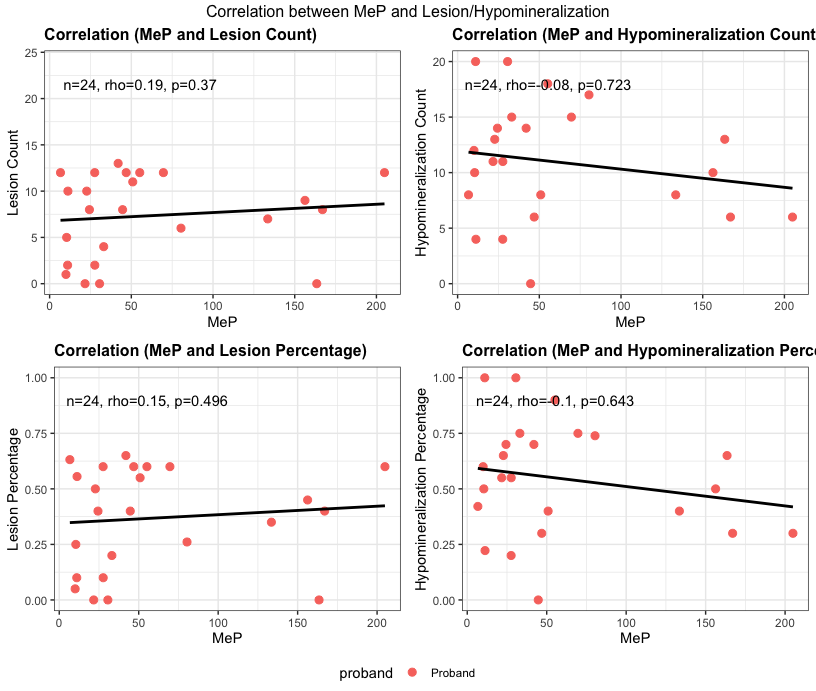


**Supplemental Figure 11. Correlation between Methylparaben (MeP) in maternal urine, and numbers of teeth with dental lesions, numbers of teeth with hypomineralization, percentage of teeth with hypomineralization, and percentage of teeth with lesions. Each dot represents one child/mother dyad.**

**Supplemental Figure 12.**


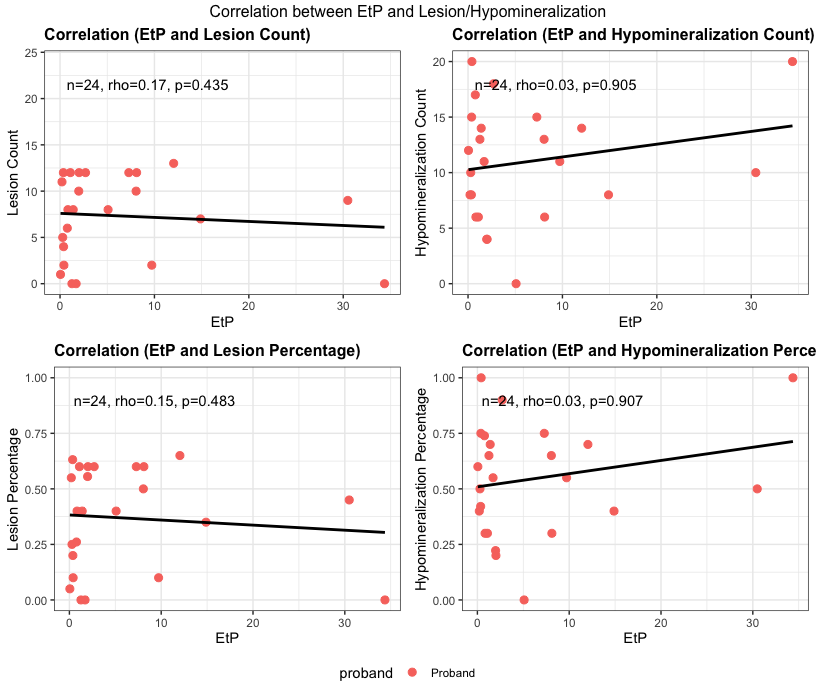


**Supplemental Figure 12. Correlation between ethyl phthalate (EtP) in maternal urine, and numbers of teeth with dental lesions, numbers of teeth with hypomineralization, percentage of teeth with hypomineralization, and percentage of teeth with lesions. Each dot represents one child/mother dyad.**

**Supplemental Figure 13.**


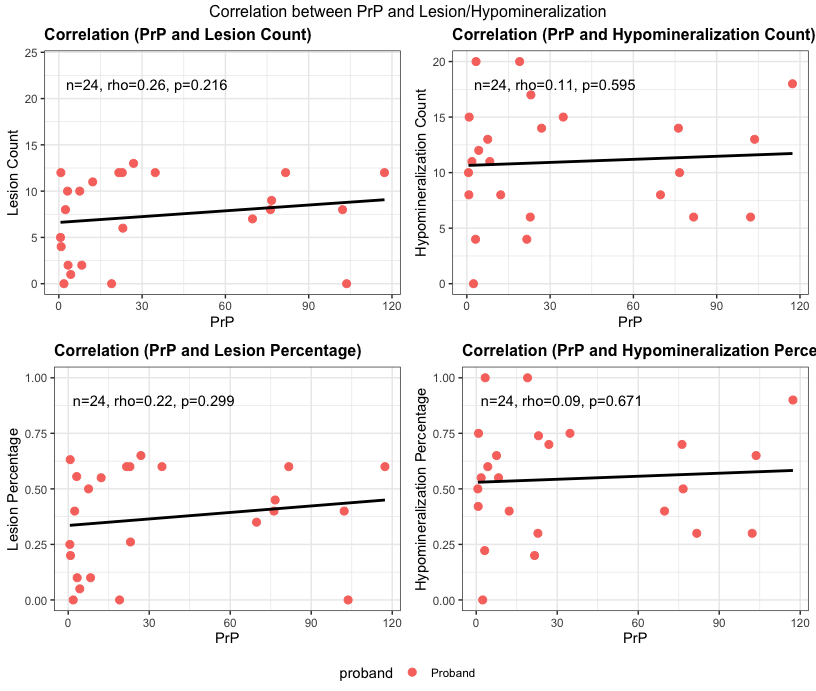


**Supplemental Figure 13. Correlation between Propylparaben (PrP) in maternal urine, and numbers of teeth with dental lesions, numbers of teeth with hypomineralization, percentage of teeth with hypomineralization, and percentage of teeth with lesions. Each dot represents one child/mother dyad.**

**Supplemental Figure 14.**


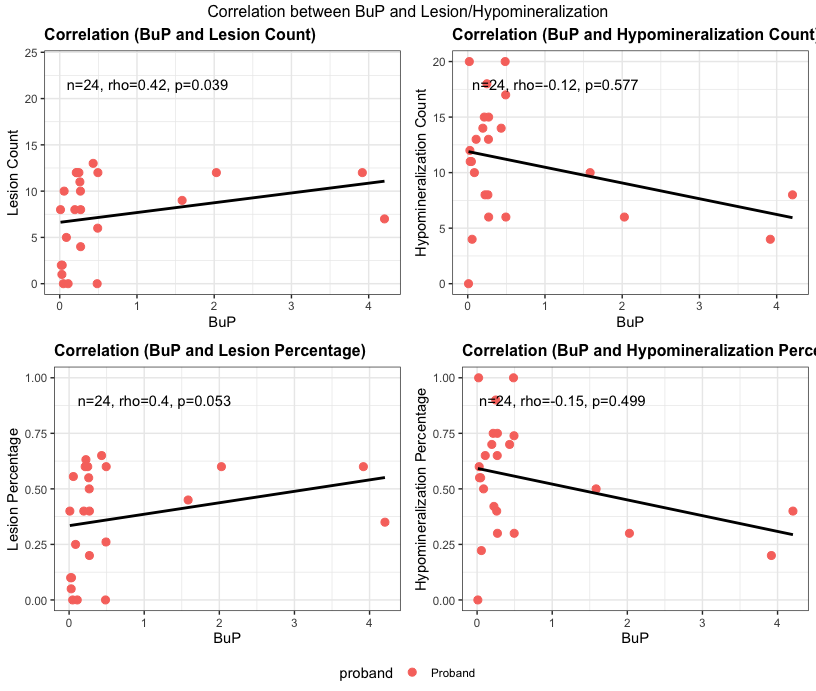


**Supplemental Figure 14. Correlation between Butylparaben (BuP) in maternal urine, and numbers of teeth with dental lesions, numbers of teeth with hypomineralization, percentage of teeth with hypomineralization, and percentage of teeth with lesions. Each dot represents one child/mother dyad.**

**Supplemental Figure 15.**


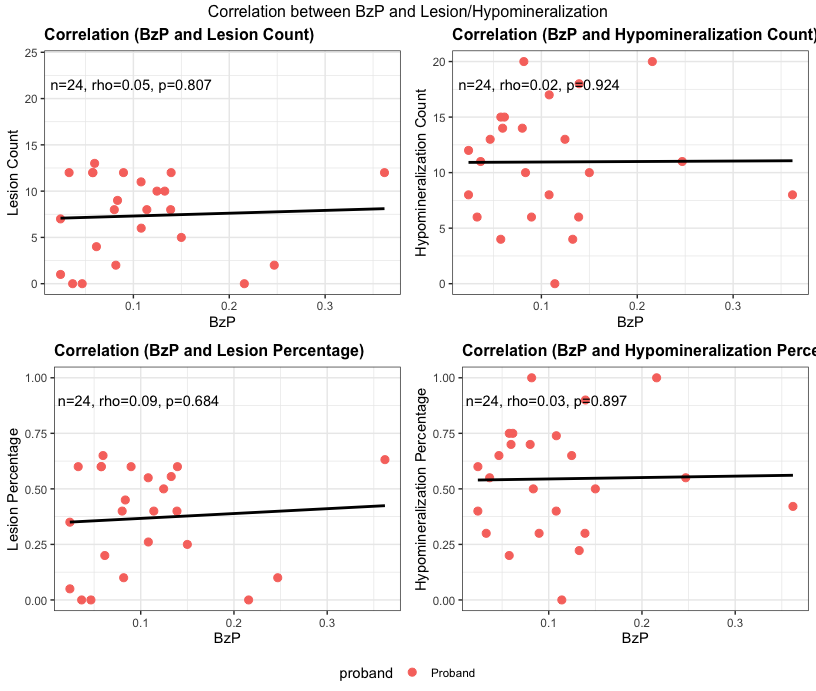


**Supplemental Figure 15. Correlation between Benzylparaben (BzP) in maternal urine, and numbers of teeth with dental lesions, numbers of teeth with hypomineralization, percentage of teeth with hypomineralization, and percentage of teeth with lesions. Each dot represents one child/mother dyad.**
